# Supplementary material for: Alignment of the metatarsal heads affects foot inversion/eversion during tiptoe standing on one leg in demi–pointe position: A cross–sectional study on recreational dancers
Source: PLoS One. 2022 Oct 18;17(10):e0276324. doi: 10.1371/journal.pone.0276324 (PMC9578639; doi:10.1371/journal.pone.0276324)
Supplement: S1 File — Positions of reflective markers on the body and anatomical labels are listed. (DOCX) [file pone.0276324.s001.docx]

**Additional Material 1.** Positions of reflective markers on the body and anatomical labels

| 1 | Right anterior superior iliac spine | 16 | Right fifth metatarsal head |
| --- | --- | --- | --- |
| 2 | Left anterior superior iliac spine | 17 | Left fifth metatarsal head |
| 3 | Midpoint between the right and left posterior superior iliac spines | 18 | Midpoint between the right second metatarsal and the right second phalange |
| 4 | Marker cluster for the calculation of the right hip joint center | 19 | Midpoint between the left second metatarsal and the left second phalange |
| 5 | Marker cluster for the calculation of the left hip joint center | 20 | Right distal phalanx of the second toe |
| 6 | Midpoint between the right lateral epicondyle and the right lateral condyle of the tibia | 21 | Left distal phalanx of the second toe |
| 7 | Midpoint between the left lateral epicondyle and the left lateral condyle of the tibia | 22 | Right first metatarsal head |
| 8 | Midpoint between the right medial epicondyle and the right medial condyle of the tibia | 23 | Left first metatarsal head |
| 9 | Midpoint between the left medial epicondyle and the left medial condyle of the tibia | Posterior side  Anterior side 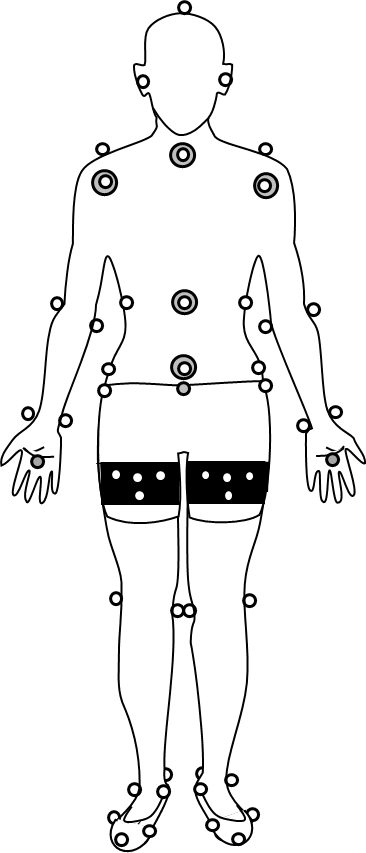 22  21  17  19  16  18  10  12  6  8  1  2  3  4  5  7  9  11  13  14  15  23  20 | |
| 10 | Right calcaneus |  |  |
| 11 | Left calcaneus |  |  |
| 12 | Right medial malleolus |  |  |
| 13 | Left medial malleolus |  |  |
| 14 | Right lateral malleolus |  |  |
| 15 | Left lateral malleolus |  |  |
