# Supplementary material for: Alignment of the metatarsal heads affects foot inversion/eversion during tiptoe standing on one leg in demi–pointe position: A cross–sectional study on recreational dancers
Source: PLoS One. 2022 Oct 18;17(10):e0276324. doi: 10.1371/journal.pone.0276324 (PMC9578639; doi:10.1371/journal.pone.0276324)
Supplement: S2 File — Measurement of the the angle between adjacent metatarsal heads using the MR images are explained in this document. (DOCX) [file pone.0276324.s002.docx]

**Additional Material 3.** Measurement of the angle between adjacent metatarsal heads. While referring to the sagittal image of the metatarsal bone, a frontal image was selected wherein the metatarsal head was in contact with the slice plane. The selected image of the toe was where the metatarsal head was first visible when the horizontal images were arranged from the sole toward the instep.


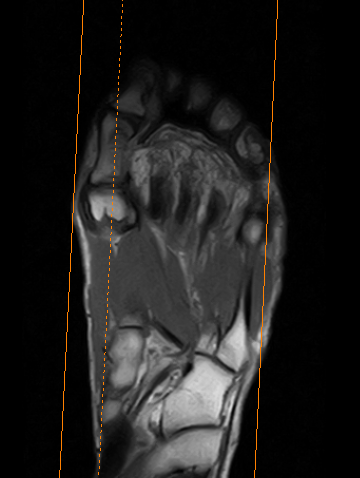

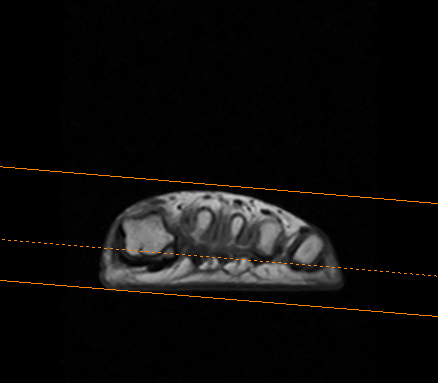

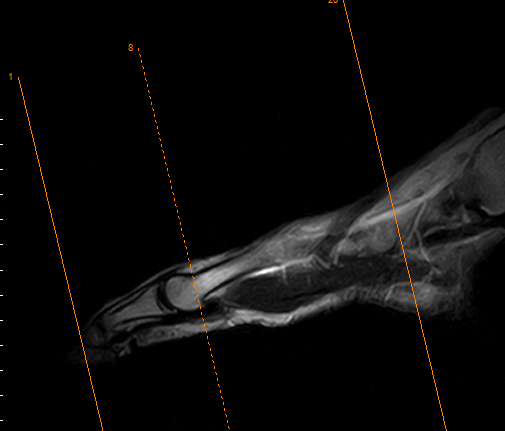


Sagittal image

Frontal image

Horizontal image

Frontal, sagittal and horizontal images of the metatarsal head when determining the grounding point of the first toe. The dashed line in the frontal image shows the slice plane of the selected horizontal image. The dashed line in the sagittal image shows the slice plane of the selected frontal image. The dashed line in the horizontal image shows the slice plane of the selected sagittal image.


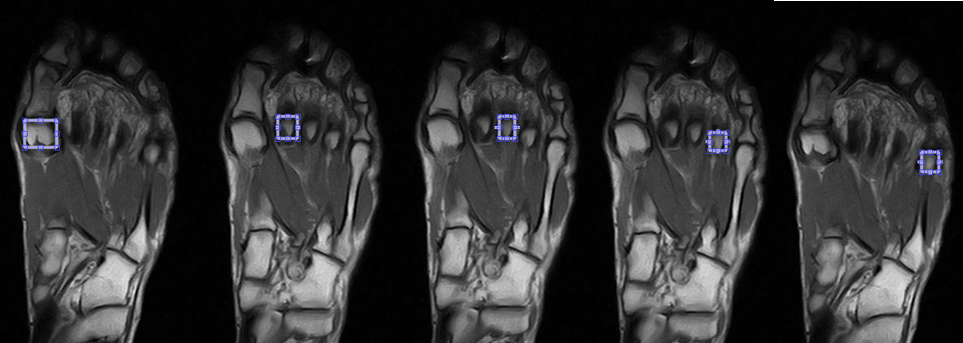


While referring to the selected frontal image, the area considered to be closest to the ground in the horizontal image was marked. The brightest point in the marked area was assumed to be the bone, and its two-dimensional coordinates were obtained. This procedure was repeated for each toe, and the angles between adjacent metatarsal heads were calculated using the coordinates of the five points obtained (Figure 2d). Because the first and fifth metatarsal heads and the second, third, and fourth metatarsal heads were each at the lowest level in the same slice planes, the same images were used.
