# Supplementary figures and images for: Alignment of the metatarsal heads affects foot inversion/eversion during tiptoe standing on one leg in demi–pointe position: A cross–sectional study on recreational dancers
Source: PLoS One. 2022 Oct 18;17(10):e0276324. doi: 10.1371/journal.pone.0276324 (PMC9578639; doi:10.1371/journal.pone.0276324)

**Additional Material 2.** Wooden plate used to prevent curling of the non-weightbearing forefoot.


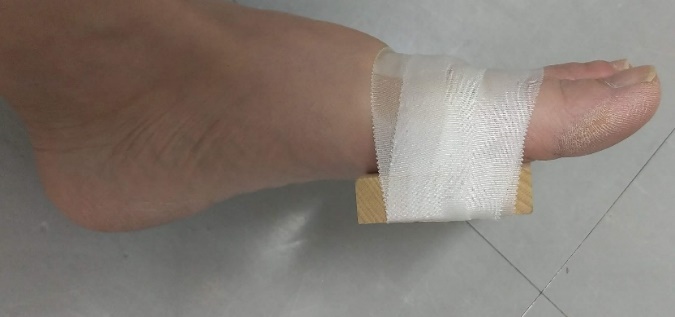

Supplement: S1 Fig — The plate was fixed on the forefoot using a surgical tape. (DOCX) [file pone.0276324.s003.docx]
