# Supplementary material for: Alignment of the metatarsal heads affects foot inversion/eversion during tiptoe standing on one leg in demi–pointe position: A cross–sectional study on recreational dancers
Source: PLoS One. 2022 Oct 18;17(10):e0276324. doi: 10.1371/journal.pone.0276324 (PMC9578639; doi:10.1371/journal.pone.0276324)
Supplement: Attachment — Submitted filename: 2022_AI_reply.docx [file pone.0276324.s004.docx]

Dear Editor,

We sincerely appreciate your taking the time to review our manuscript. We have checked the journal requirements according to the comments from Reviewer 1. We replied to the comments point by point as follows;

Journal Requirements:

When submitting your revision, we need you to address these additional requirements.

1. Please ensure that your manuscript meets PLOS ONE's style requirements, including those for file naming. The PLOS ONE style templates can be found at

https://journals.plos.org/plosone/s/file?id=wjVg/PLOSOne_formatting_sample_main_body.pdf and

https://journals.plos.org/plosone/s/file?id=ba62/PLOSOne_formatting_sample_title_authors_affiliations.pdf

**

We checked the above files and modified the manuscript to meet the style requirements.

2. Thank you for stating the following financial disclosure:

"No"

At this time, please address the following queries:

a) Please clarify the sources of funding (financial or material support) for your study. List the grants or organizations that supported your study, including funding received from your institution.

b) State what role the funders took in the study. If the funders had no role in your study, please state: "The funders had no role in study design, data collection and analysis, decision to publish, or preparation of the manuscript."

c) If any authors received a salary from any of your funders, please state which authors and which funders.

d) If you did not receive any funding for this study, please state: "The authors received no specific funding for this work."

Please include your amended statements within your cover letter; we will change the online submission form on your behalf.

**

We have included the answers to comments a-c in the cover letter.

3. In your Data Availability statement, you have not specified where the minimal data set underlying the results described in your manuscript can be found. PLOS defines a study's minimal data set as the underlying data used to reach the conclusions drawn in the manuscript and any additional data required to replicate the reported study findings in their entirety. All PLOS journals require that the minimal data set be made fully available. For more information about our data policy, please see http://journals.plos.org/plosone/s/data-availability.

"Upon re-submitting your revised manuscript, please upload your study's minimal underlying data set as either Supporting Information files or to a stable, public repository and include the relevant URLs, DOIs, or accession numbers within your revised cover letter. For a list of acceptable repositories, please see http://journals.plos.org/plosone/s/data-availability#loc-recommended-repositories. Any potentially identifying patient information must be fully anonymized.

Important: If there are ethical or legal restrictions to sharing your data publicly, please explain these restrictions in detail. Please see our guidelines for more information on what we consider unacceptable restrictions to publicly sharing data: http://journals.plos.org/plosone/s/data-availability#loc-unacceptable-data-access-restrictions. Note that it is not acceptable for the authors to be the sole named individuals responsible for ensuring data access.

We will update your Data Availability statement to reflect the information you provide in your cover letter.

**

We included the DOIs of the minimal data set in the cover letter.

4. Please amend either the title on the online submission form (via Edit Submission) or the title in the manuscript so that they are identical.

**

The title listed in the manuscript and that on online submission form were verified and are identical.

5. Please review your reference list to ensure that it is complete and correct. If you have cited papers that have been retracted, please include the rationale for doing so in the manuscript text, or remove these references and replace them with relevant current references. Any changes to the reference list should be mentioned in the rebuttal letter that accompanies your revised manuscript. If you need to cite a retracted article, indicate the article's retracted status in the References list and also include a citation and full reference for the retraction notice.

**

We have added three new references in the revised manuscript in response to the reviewer's comments. We mentioned all the references in the manuscript.

*****************************************************************************************

Reviewer #1: Summary

The research project investigate the relationship between metatarsal alignment in both load positions and foot inversion/eversion angle during tiptoe standing. Authors hypothesized that the angles between adjacent metatarsal heads are related to foot inversion/eversion and to the proportion of load on the medial and lateral of the foot; Authors also hypothesized these relationships are different between both legs. 9 ballet dancers are collected with experimental design.

**

We would like to express our appreciation to the reviewer for clear understanding of our

study and the helpful suggestions for the improvement of our manuscript. We agree with your comments and have modified our manuscript have revised our manuscript in line with your comments.

We reconsidered adding explanations about foot inversion/eversion. This study aimed to examine why the supporting foot might turn inward or outward when standing on tiptoe with a single leg. While this foot rotation is called foot sickling–out or sickling–in in dance, non–dance people may be unfamiliar with this word. Therefore, we named it foot inversion/eversion in the manuscript. We mentioned this in the revised manuscript in Lines 53-57.

Major issues.

Some of figures need clarification. Figure 1 could be improved to emphasize the toe extension and ankle plantarflexion.

**

We added an enlarged photo of the foot during tiptoe standing, as shown in Fig 1b. Also, we added the angle of AG_IE, the variable presenting the foot inversion/eversion, in Fig 1c.

Does Figure 2 represent the average dancer's metatarsal heads? Where the image source came form? As this image provides a great platform explaining research questions, clear source of images might be needed.

**

Figure 2 represents MR images of a dancer in this study.

It is also not clear how information derived from the results in Figure 3. I suggest the authors need to provide clear explanation in each box.

**

To find the results of Figures 3 and 4 quickly, we arranged the contents of the Results section, the tables, and the figures (Lines 231-288) and added simple explanations to each box in the caption (Figs 3 and 4).

To track how to determine these variables quickly, we added the abbreviations used in Figures 3 and 4 into sentences which explain how to determine the variables (Lines 170 & 186). Furthermore, we divided the Data analysis section in the Method into two sub–sections, "Kinematics and kinetics during tiptoe standing" and "Measuring angles between metatarsal heads on MR images".

Minor issues

The authors should check whether there is a reporting guideline suitable for their study; usually, there will be and re-write the manuscript following a guideline checklist. It offers a standard way for authors to prepare report of trial findings, facilitating their complete and transparent reporting, and aiding their critical appraisal and interpretation.

**

We referred to the guideline for analysing kinematics and kinetics by [21] Derrick et al. (2020) and [22] Leardini et al. (2021). We also referred to [23] Weishaupt et al. (2002), as they measured the distance between the bottom of the metatarsal head bone and the sole using MR images.

The main limitation is the 9 participants as authors explained in the limitation section, still it should be justified in the text why it is "okay" to conclude your hypotheses with small number of subjects.

**

The normality of distributions and a type two error are concerns when the sample number is small. We tested the variables after verifying the normality of distribution, and then parametric or non–parametric tests were used accordingly to obtain the accurate p–values. However, the type two error might still occur. Therefore, we added sentences about the type two error caused by the small number of participants, especially in interpreting the limitation of leg kinematics and kinetics results (Lines 345-348). Furthermore, we limited the interpretation of the results to recreational dancers in the title and Conclusion (Line 367).

The methodology is not clear enough. For example, I don't see any information about motion marker-data collection part. This is a fairly significant omission (or am I missing some part?). Methods to Data analysis should be linked, but this does not seem to be reported in the manuscript.

**

We have added a more detailed explanation of motion capturing according to the guideline for analysing kinematics and kinetics (Lines 133-142). The additional material (S1 File) provides the information on the marker set.
